# Dr. Alfred W. Henckell

**Published:** 1912-08

**Authors:** 


Dr. Alfred W. Henckell, Buffalo, 1889, died June 30, at his home 529 West Ave., Rochester, aged 46. He leaves a wife, Mrs. Ida Orphy Henckell; two daughters, Esther Henckell, aged 13, and Marion Henckell, aged 12; a mother, Mrs. Rosalie A. Henckell of 66 Lenox Street, Rochester; two brothers, Carl Henckell of Birmingham, Ala., and William Henckell of Silver Springs, Md., and a sister, Mrs. H. F. Galvin of Boston. He was a son of the late Rev. Emil Henckell, who was pastor of the German United Evangelical Trinity Church in Allen Street.

Dr. Henckell was born in Germany in 1866 and his parents came to America when he was 4 years old. He was educated in the common schools and the old Free Academy. He studied medicine in the University of Buffalo and was graduated there in 1889, at the head of his class. He went abroad for study and travel for one year and returned to Rochester, where he began his practice in 1890. He had been in continuous and successful practice since then.

In 1896 Dr. Henckell was married to Miss Ida Orphy. He was a prominent member of the Masonic fraternity, a member of Monroe Commandery, Knights Templar, and of Damascus Temple of the Nobles of the Mystic Shrine. He was a member of the American Medical Association, New York State Medical Society, Central New York Medical Association, Monroe County Medical Association, Rochester Pathological Society and Rochester Academy of Medicine.

